# Analysis of Urban Heat Island and Heat Waves Using Sentinel-3 Images: a Study of Andalusian Cities in Spain

**DOI:** 10.1007/s41748-021-00268-9

**Published:** 2021-11-02

**Authors:** David Hidalgo García

**Affiliations:** Technical Superior School of Building Engineering, University of Granada, Fuentenueva Campus, 18071 Granada, Spain

**Keywords:** Surface Urban Heat Island, Heat waves, Sentinel-3 imagery, Land surface temperature, Heat Resilience and Urban Resilience

## Abstract

**Abstract:**

At present, understanding the synergies between the Surface Urban Heat Island (SUHI) phenomenon and extreme climatic events entailing high mortality, i.e., heat waves, is a great challenge that must be faced to improve the quality of life in urban zones. The implementation of new mitigation and resilience measures in cities would serve to lessen the effects of heat waves and the economic cost they entail. In this research, the Land Surface Temperature (LST) and the SUHI were determined through Sentinel-3A and 3B images of the eight capitals of Andalusia (southern Spain) during the months of July and August of years 2019 and 2020. The objective was to determine possible synergies or interaction between the LST and SUHI, as well as between SUHI and heat waves, in a region classified as highly vulnerable to the effects of climate change. For each Andalusian city, the atmospheric variables of ambient temperature, solar radiation, wind speed and direction were obtained from stations of the Spanish State Meteorological Agency (AEMET); the data were quantified and classified both in periods of normal environmental conditions and during heat waves. By means of Data Panel statistical analysis, the multivariate relationships were derived, determining which ones statistically influence the SUHI during heat wave periods. The results indicate that the LST and the mean SUHI obtained are statistically interacted and intensify under heat wave conditions. The greatest increases in daytime temperatures were seen for Sentinel-3A in cities by the coast (LST = 3.90 °C, SUHI = 1.44 °C) and for Sentinel-3B in cities located inland (LST = 2.85 °C, SUHI = 0.52 °C). The existence of statistically significant positive relationships above 99% (*p* < 0.000) between the SUHI and solar radiation, and between the SUHI and the direction of the wind, intensified in periods of heat wave, could be verified. An increase in the urban area affected by the SUHI under heat wave conditions is reported.

**Graphical Abstract:**

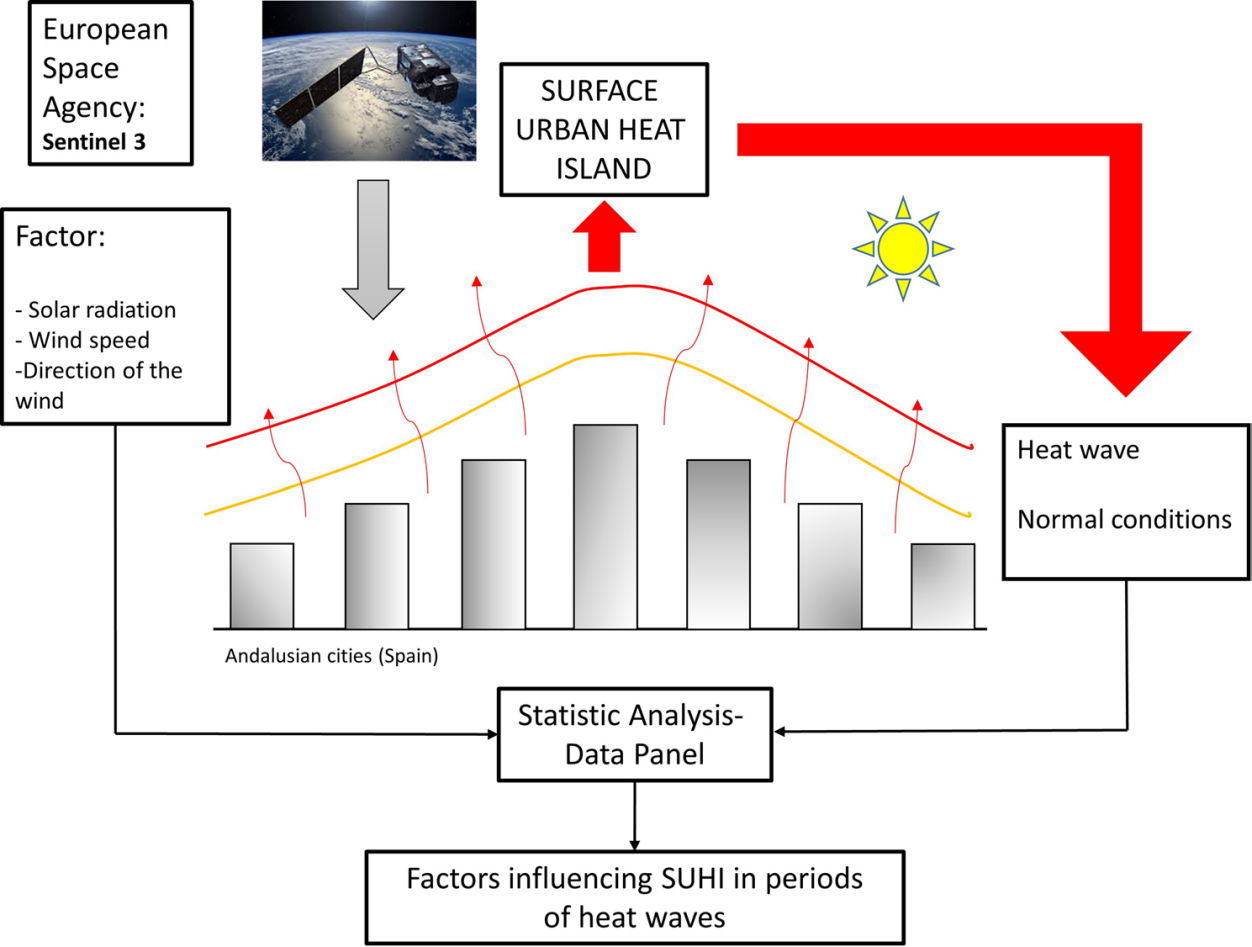

**Supplementary Information:**

The online version contains supplementary material available at 10.1007/s41748-021-00268-9.

## Introduction

In recent decades, numerous studies warn that the transformation of the landscape owing to the expansion of urban areas is one of the processes that contributes most to climate change (Li et al. 2011; Carvalho et al. 2017; Jiang et al. 2019; Yang et al. 2019; Song et al. 2020). Changes in land cover increase the surfaces of impermeable materials, such as asphalt and concrete, reducing evapotranspiration (Stewart and Oke 2012). These materials are known to store the heat coming from solar radiation and subsequently release it into the atmosphere (Arnfield 2003; Zhou et al. 2015; An et al. 2020).

The greatest increases in temperature occur in cities, mainly due to a phenomenon of urban climate alteration (Li et al. 2011; Zhou et al. 2015; Wang et al. 2016; Luo and Lau 2018; Zhao et al. 2018; Tewari et al. 2019; Anjos et al. 2020) called Urban Heat Island (UHI), whose intensity is heightened by multiple human activities (Lai et al. 2018; Huang et al. 2020; Santamouris 2020) and by extreme weather events such as droughts or heat waves. The positive interaction between UHI and heat waves is well documented: in Baltimore and Maryland (Li and Bou-Zeid 2013), Beijing (Li et al. 2015; Jiang et al. 2019), New York (Ramamurthy and Bou-Zeid 2017), Shanghai and Guangzhou (Jiang et al. 2019) and Athens (Founda and Santamouris 2017).

Research shows that heat waves are becoming more intense, lasting longer, and occurring more frequently (Meehl and Tebaldi 2004; Sun et al. 2014). It is anticipated that by the end of the twenty-first century they will affect larger land areas (Meehl and Tebaldi 2004; Lau and Nath 2012; Coumou et al. 2013). Episodes of increased anthropogenic heat are known to be among the natural phenomena having the greatest social, economic and environmental impact (An et al. 2020). They imply more consumption of electricity and water in homes (Valor et al. 2001), and increased morbidity and mortality (Semenza et al. 1996; Poumadère et al. 2005; Jiang et al. 2019; An et al. 2020). Proof can be found in the heat wave of Chicago in 1995, which caused 800 deaths (Semenza et al. 1996), that of the summer of 2003 in Europe, when 70,000 people died (Robine et al. 2008), the one occurring in Russia during the summer of 2010 (Grumm 2011), that of eastern China in 2013 (Xia et al. 2016), or Northwestern USA and Western Canada (Lytton) in 2021, with temperatures over 45.0 °C on consecutive days and extremely warm nights in between, causing some 500 deaths (UNO 2021).

While a positive interaction between SUHI and heat waves has been demonstrated, the type of climate or particular climatic conditions (wind speed and direction, solar radiation) and geomorphological factors of cities are contribute substantially to this interaction (Zhao et al. 2014; Yoon et al. 2018; Jiang et al. 2019; An et al. 2020; Qiu et al. 2020; Venter et al. 2020). Studies of cities in Oklahoma (Basara et al. 2010), several European cities (Founda et al. 2015), London (Gregor et al. 2007) or Beijing and Guangzhou (Jiang et al. 2019) report increases in SUHI that are stronger during the night. In contrast, studies of Athens and Parma (House and Santamouris 2011) and Shanghai (Ao et al. 2019; Jiang et al. 2019) found that the SUHI rise was stronger during the day. Other studies found no significant amplification of the SUHI (Ramamurthy and Bou-Zeid 2017; Scott et al. 2018; Zhao et al. 2018).

Among the different methodologies used to determine this phenomenon, thermal remote sensing stands out because of its capacity to allow large-scale urban studies of LST and SUHI using satellite images with Thermal Infrared Sensor (TIRS) sensors. Studies involving these systems and the dynamics of urban climate have become consolidated as an important field of research (Ramamurthy and Bou-Zeid 2017) with an extensive body of literature (Wang and Ouyang 2017; Song et al. 2018; Yao et al. 2018; Sejati et al. 2019; Guo et al. 2020; Hu et al. 2020; Roy et al. 2020; Shafizadeh et al. 2020; Yang et al. 2020a). A relatively recent but highly accurate product used in many studies is Sentinel-3 imaging. All Sentinels have 3 TIRS channels—bands 7, 8 and 9—that provide LST estimates at a resolution of 1000 m. Their use for this type of research lends an important advantage over satellites such as Landsat or NOAA, since they orbit twice a day over the same point on the planet—once during the day and again at night. The use of Sentinel-3 is widely documented in the literature, e.g., through SUHI studies of the cities of Daman (India) and Huazhaizi (China) (Yang et al. 2020b), Oklahoma City (USA) and Dahra (Senegal) (Sobrino et al. 2016).

The space–time variability of the SUHI in cities under Heat Wave conditions is largely unknown, and very few studies have focused on cities in the Mediterranean Basin. Recent estimates are that the mean air temperature will be 1–3 °C higher in the near future (compared to 1961–1990), 3–5 °C higher by the middle of the century (2040–2069) and approximately 3.5–7 °C higher by the end of the century (2070–2100) (Founda et al. 2015; Founda and Santamouris 2017); values for the Mediterranean Sea basin may be even higher (Ward et al. 2016; Cramer et al. 2018).

The fact that temperature in the Mediterranean region is increasing at a faster rate than elsewhere in the world leads it to be considered an area of high vulnerability due to climate change. Such potentially dire circumstances, together with the variability of the data, accentuate the need for detailed research efforts. In this case, a quantitative and systematic study of the existence of synergies between SUHI and heat waves in the cities of Andalusia (Spain) was undertaken.

This adverse meteorological phenomenon is a problem that tends to affect urban populations in particular. The synergies between SUHI and heat waves may be questioned, however, owing to disparate results reported to date, and insufficient knowledge about the factors affecting their intensity, properties, and activation flow. Such information is crucial for the establishment of adequate mitigation or resilience measures for urban planning in attempts to limit the effects and economic cost of heat waves (Emmanuel and Krüger 2012). This research aims to analyze the relationship between heat wave and three outstanding factors: solar radiation, wind speed and direction.

Our study was intended to characterize and quantify the variability of the LST and the day and night SUHIs of all eight Andalusian (southern Spain) capital cities using Sentinel-3 images, throughout the months of July and August of 2019 and 2020, when five heat waves occurred. The factors involved were statistically analyzed using the Data Panel method. The methodology entailed an open source environment allowing one to monitor SUHI the variations in a precise, urgent and economic way, providing for a more comprehensive understanding of the space–time variability of the SUHI during Heat Wave periods, and of underlying factors.

## Materials and Methods

### Study Area and Data Source

**Fig. 1:**
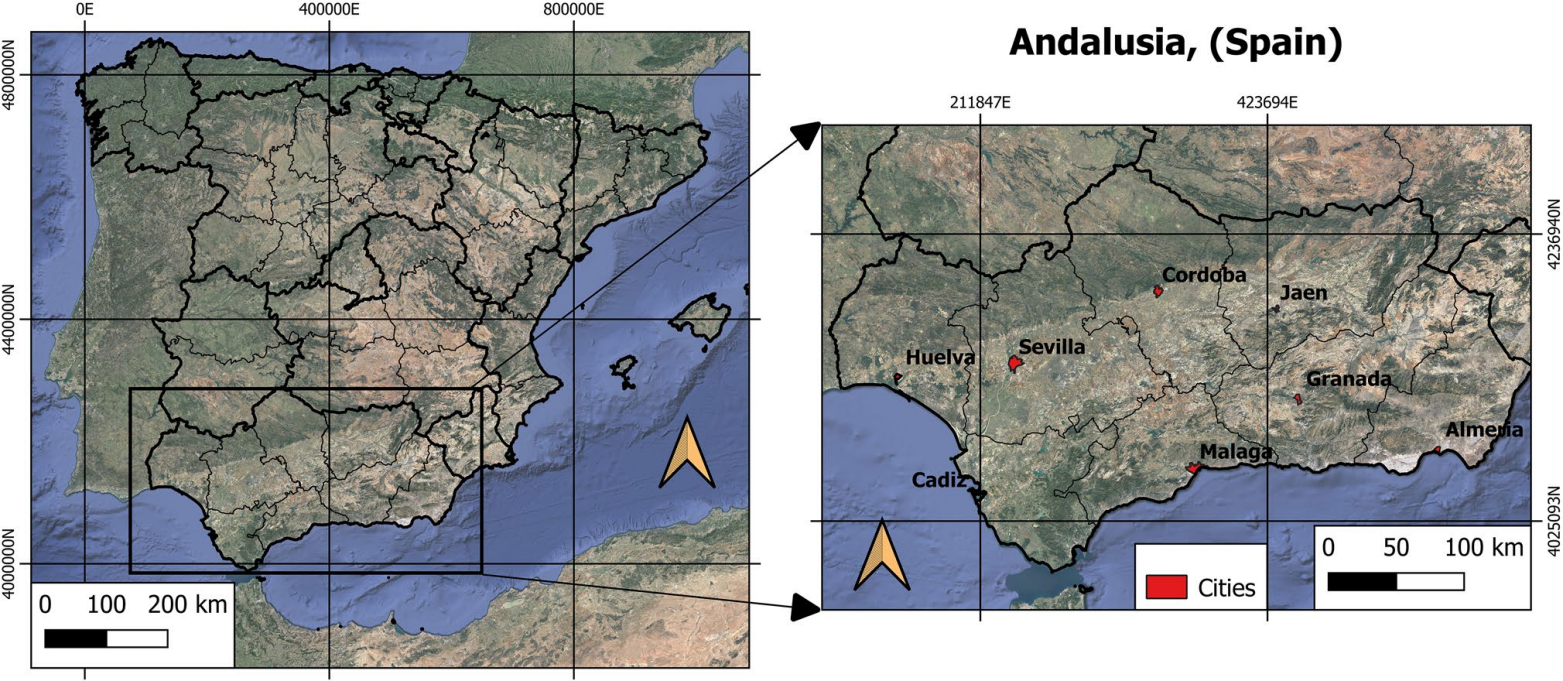
Study area, Andalusia, Spain

The area under study comprises the eight provincial capitals of the region of Andalusia, located in southern Spain (Fig. 1).

Four of them are inland cities: Sevilla, Cordoba, Granada and Jaen. The other four are coastal cities: Huelva, Cadiz, Málaga and Almería. Characteristics of the population, surface area, climate, rainfall, altitude and UTM coordinates are found in Table 1.

**Table 1:** Characteristics of inland cities of Andalusia

Geographic information	Inland cities				Coastal cities			
	Sevilla	Cordoba	Jaen	Granada	Huelva	Cadiz	Malaga	Almeria
Downtown location UTM	37.375 N, − 6.025 W	37.891 N, − 4.819 W	37.780 N, − 3.831 W	37.111 N, − 3.362 W	37.270 N, − 6.974 W	36.516 N, − 6.317 W	36.765 N, − 4.564 W	36.841 N, − 2.492 W
Climate Zone	Csa	Csa	Csa	Csa—Bsk	Csb	Csb	Csa	Bsk
Mean annual T. (°C)	18.6	17.8	16.9	15.5	17.8	17.9	18.4	17.9
Average annual rainfall (mm)	576	612	552	450	467	597	520	228
Total area (km^2^)	140.8	1253	424	88.8	151.3	13.3	398	296.2
Total urban area (km^2^)	68.69	31.35	9.43	21.78	14.87	7.34	58.6	14.95
Population in 2019 (hab)	688,592	325,701	112,999	232,462	143,663	116,027	574,654	198,533
Urban mean elevation (masl)	11	106	570	680	24	13	8	16

Climate Zones: *Csa* Mediterranean Climate, *Csb* Mediterranean Oceanic Climate, *Bsk* Cold Semi-Arid Climate

According to Spain´s National Institute of Statistics (INE), Andalusia covers an area of 87,268 km^2^ and has a population of 8,427,325, being the second largest region and the most populated one in all of Spain. The region shows different local background climates. According to the Koppen-Geiger climate classification, the cities of Cadiz and Huelva share a Mediterranean Oceanic climate (Csb), the cities of Sevilla, Malaga, Cordoba and Jaen feature a Mediterranean climate (Csa), and Granada and Almería have a cold semi-arid climate (Bsk). Such typologies imply mild, humid winters and hot, dry summers (De Castro et al. 2007). The region is bordered by mountains to the north, while the Mediterranean Sea lies to the south. This circumstance makes the sea and land breezes strongly impact coastal cities. The average altitude is 503 m above sea level; the annual average temperature fluctuates between 11 °C in January and 26.5 °C in July, with minima in winter of − 3 °C and extremes in summer of 44 °C. The approximate number of hours of sunshine per year ranges between 2800 and 3200, giving an average between 7.67 and 8.76 h of sunshine per day, depending on the area within the Andalusian region.

### Methodology

**Fig. 2:**
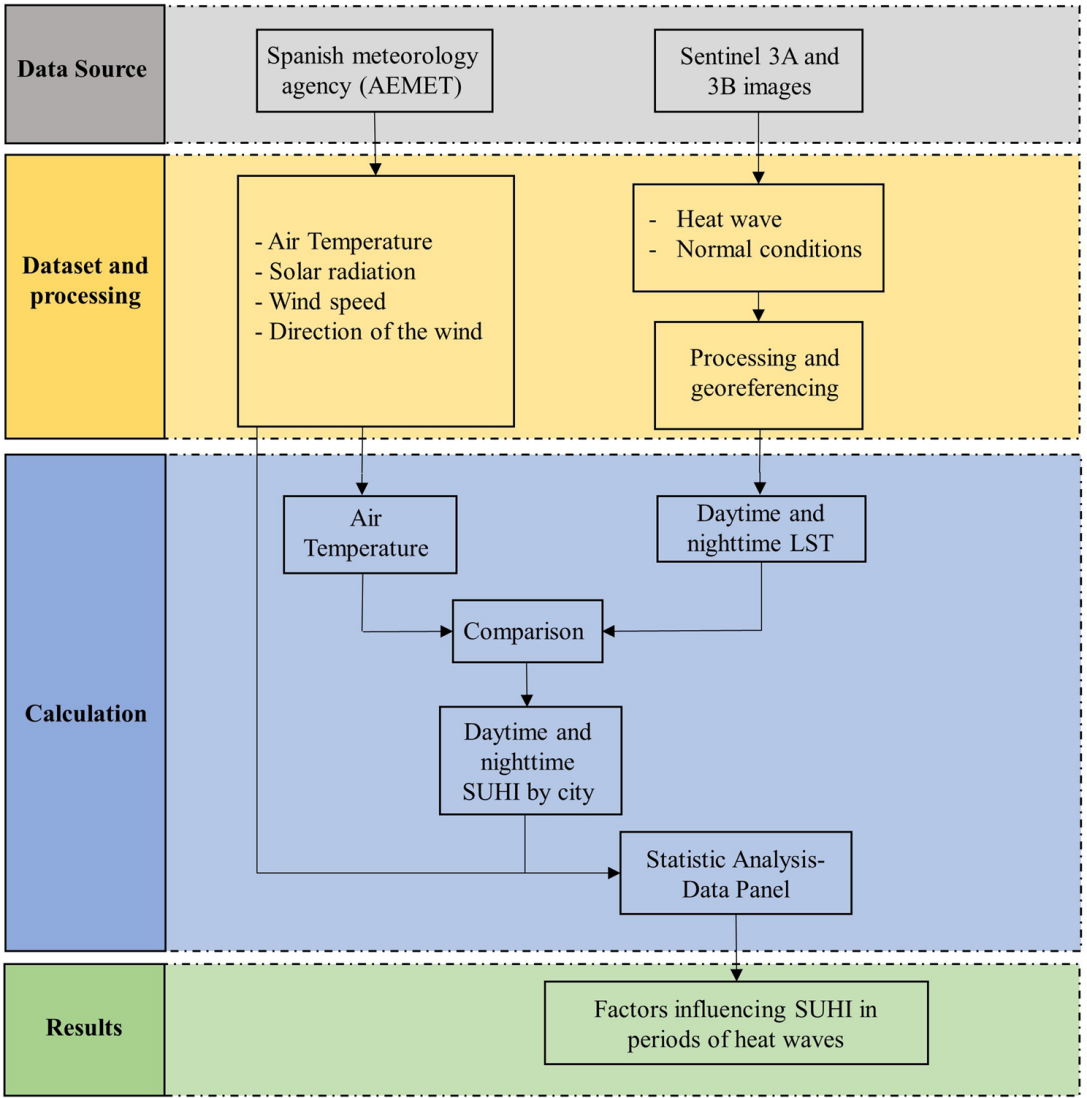
Methodology of our research

The methodology carried out in this research called for obtaining the LST using Sentinel-3 images and validating them by comparison with the ambient temperatures recorded by AEMET (Srivastava et al. 2009; Gallo et al. 2011; Li et al. 2013; Avdan and Jovanovska 2016; Rongali et al. 2018). They were classified in periods of normal environmental conditions and in periods under heat wave. Next, the LST and SUHI values were obtained for statistical analysis, as seen in Fig. 2.

The Data Panel statistical method was used for data analysis. Unlike more traditional methods of analysis, it admits a greater number of data, including the individual effects of each city in the overall result, while eliminating the problem of collinearity between variables. Using this method allowed us to reflect possible variations in the conditions of each city contemplated in the final results, which makes it a unique and powerful approach. It has been validated by studies (Chen et al. 2011; Alcock et al. 2015; Fang and Tian 2020) similar to ours, accounting for time series of multiple cities or areas, as well as quantitative variables when the conditions may vary among the cities analyzed.

### Identification of Heat Waves

According to the AEMET, during 2019 three episodes classified as heat waves in Andalusia, and in 2020 just two. Table 2 indicates their onset, end date and duration, along with the thermal anomaly they produced in room temperature and the maximum temperature reached. Although numerous studies cite decreased environmental pollution, LST and SUHI as a consequence of the lockdown situation caused by COVID-19 (Ali et al. 2021; Das et al. 2021; Ghosh et al. 2020; Jiang et al. 2021; Mandal and Pal 2020; Nakajima et al. 2021; Pani et al. 2020; Srivastava et al. 2021; Toro et al. 2021), no scientific evidence stands to indicate a decrease in heat waves due to or during this situation.

**Table 2:** Characteristics of the heat waves studied in Andalusia

Heat waves	2019			2020	
	1st	2nd	3rd	4th	5th
Start date	26/06/2019	20/07/2019	06/08/2019	30/07/2020	05/08/2020
End date	01/07/2019	25/07/2019	10/08/2019	01/08/2020	08/08/2020
Duration (days)	6	6	5	3	4
Air thermal anomaly (C)	4	2	3.3	4	5
Maximum air temperature reached (C)	38.8	36.8	37.9	38.5	39.4

Source: State Meteorological Agency (AEMET)

To facilitate comparison of the LST and SUHI of periods under heat wave conditions with periods of “normal” environmental conditions, the 2 days before and after each heat wave period were taken into account. In total, the environmental parameters of the eight cities were studied for 20 days under normal conditions and 24 days under heat wave conditions.

### Sentinel-3 Images. Land Surface Temperature Estimation

Sentinel-3 satellites are equipped with the high-resolution scanning instrument LST Radiometer, enabling LSTs of the Earth's surface to be obtained. Its thermal products have three levels of processing (levels 0, 1 and 2), although only the last two are available for download. Those of level 1 present radiance and brightness temperatures that require split window (SW) algorithms to obtain the LST. Level 2 products directly and automatically include the LST together with associated parameters such as the Normalized Vegetation Index (NDVI), Vegetation Type (Biome), Vegetable Fraction (Pv) and Normalized Difference Index (NDBI).

The existing SW algorithms that serve to gauge LST are based on the concept of differential absorption (McMillin 1975), whereby the difference between the two TIRS band wavelengths allows for correction of the atmospheric effects produced on the signal. Abundant studies report on the validation, use and precision of these algorithms in Sentinel-3 images (Coppo et al. 2010; Wan 2013; Ruescas et al. 2016; Sobrino et al. 2016; Prikaziuk and van der Tol 2019; Chiang and Ivan 2020; Yang et al. 2020b).

The SW algorithm of the official Sentinel-3A and 3B level 2 SLSTR product implicitly incorporates soil emissivity by means of the following equation (Remedios and Emsley 2012):

1 $$LST={a}_{f,i,pw}+{b}_{f,i }{({T}_{11}-{T}_{12})}^{\frac{1}{\mathrm{cos}\left(\frac{\theta }{m}\right)}}+\left({b}_{f,i}+{c}_{f,i}\right) {T}_{12}-273.15,$$

where LST is the surface temperature in degrees C; a, b and c are coefficients dependent on the vegetation cover and the biome; and T_11_ and T_12_ are the brightness temperatures of bands 8 and 9 of Sentinel-3, respectively. θ is the zenith angle of view of the satellite and m is a dependent variable of θ (Remedios and Emsley 2012; Yang et al. 2020a).

Andalusia lies below the route of the Sentinel-3A and 3B satellites. The usual daytime hours of passage over the region are between 9:00 and 11:00 a.m.; nighttime passage is between 20:00 and 22:00 h (8:00–10:00 p.m.). The images chosen for the study correspond to 44 days in the months of July and August of 2019 and 2020. Throughout this time interval, a total of 88 images were used, 44 corresponding to Sentinel-3A (day) and 44 corresponding to Sentinel-3B (night). All of them have a cloudiness index of less than 15% to ensure accuracy in obtaining the LST and subsequently calculate the SUHI. The images used were acquired through the European Space Agency (ESA) Copernicus Open Access Hub for level 2.

After downloading the images, they were reclassified and corrected using the Toolbox (S3TBX) under the Sentinel Application Platform (SNAP) open-source software environment, version 7.0.0. With the help of SNAP 7.0.0 and using level 2 products, the day and night LST of each investigated day were recovered for each city. The LST images were subsequently exported in Geotiff format to QGIS open-source software, version 3.10.5.

### Rural Stations and Meteorological Data

The ambient temperature was obtained from AEMET. This national weather agency has multiple rural observation stations in Andalusia that hourly collect the environmental parameters of the site where they are located. The ambient temperature was needed to subsequently validate the satellite data, as indicated in the methodology section. So as to minimize the impact of the rural environment on calculation of the SUHI with Sentinel-3 images, the ones located in rural areas—surrounded by farmland and with few impervious surfaces—were selected for each city studied. This selection criterion has given statistically significant impacts in similar investigations (Wang et al. 2017; Jiang et al. 2019).

The rural stations of reference were selected taking into account the following considerations (Wang et al. 2017): (1) The % of impervious surfaces around the station is roughly 10% and the proportion of farmland must be greater than 65%; (2) the difference in surface elevation between the station and the city would be approximately 30 m; (3) rural stations had to be outside the main urban areas; (4) An approximate area of 1000 × 1000 m^2^ of equal coverage should surround the station. Given these prerequisites, a rural meteorological station was chosen for each city, its characteristics and location shown in Table 3.

**Table 3:** Characteristics of rural meteorological stations in inland cities

Geographic information	Inland cities				Coastal cities			
	Sevilla	Cordoba	Jaen	Granada	Huelva	Cadiz	Malaga	Almeria
Name of the rural temperature station	Sevilla Airport	Cordoba Airport	Jaen City	Granada Airport	Huelva City	Rota Naval base	Malaga Airport	Almeria Airport
Distance from the station to the city center (Km)	8.2	10.8	3	16	4	8.5	7	12
Impervious surface nearby (%)	16	12	15	10	23	20	15	3
Altitude (masl)	34	90	580	567	19	2	5	21
UTM	37.250 N, − 5.524 W	37.505 N, − 4.504 W	37.463 N, − 3.483 W	37.112 N, − 3.472 W	37.164 N, − 6.544 W	36.300 N, − 6.195 W	36.395 N, − 4.285 W	36.50 N, − 2.212 W

Source: State Meteorological Agency (AEMET)

Heat waves in Spain are often associated with strong anticyclonic conditions and large-scale subsidence with warm advection from North Africa in the lower atmosphere (Xoplaki et al. 2003). For the days and hours selected in this research, and from each rural meteorological station, the following data were obtained: ambient temperature, solar radiation, wind speed and direction.

Previous research (van Hove et al. 2015; Gaur et al. 2018; Jiang et al. 2019) indicates that solar radiation and wind speed and direction are elements that condition the intensity of SUHI in cities. The high pressures associated with heat waves decrease wind speed and cloud cover, which causes the earth's surface to receive more solar radiation. An increase in solar radiation produced by high pressure and low cloud cover increases environmental temperatures. Such circumstances reduce cooling and amplify the SUHI phenomenon (Oke 1987; Ackerman and Knox 2012; Li and Bou-Zeid 2013). Accordingly, certain studies (De Boeck et al. 2010; Wang et al. 2017; Jiang et al. 2019) report that during heat wave periods, solar radiation may be 2.5 times higher than under normal conditions, a fact related to SUHI amplification in many cities.

### Surface Urban Heat Island estimation

In the literature, UHI and SUHI are defined in terms of different temperatures measured within an urban area and in rural areas surrounding the city, taken at the same time (Oke 1987). UHI refers to ambient temperatures and SUHI to terrestrial surface temperatures. Therefore, the SUHI can be determined according to Eq. 2:

2 $${\text{SUHI}} = {\text{ LST}}_{{{\text{urban}}}} {-}{\text{ LST}}_{{{\text{rural}}}} .$$

Having exported the LST images of Sentinel day and night to QGIS software, version 3.10.5, and with the help of the raster calculator command, the SUHI of the city was determined by means of Eq. 2.

### Analytical Strategy

Introducing the Data Panel method of statistical analysis in the model entailed two phases (Chen et al. 2011). Firstly, by means of the Hausman proof, the effects of analysis were determined to be either fixed or random. Then the model was assessed in view of the results obtained in Wooldridge and Wald Tests. There are three options for calculation: Method of Ordinary Squares (MOS), Generalized Least Squares (GLS) and the Method of Intragroup Estimators (MIE) (Labra 2014).

The first of the three, while widely used for years, does not enable the effects of every individual to be analyzed over the course of time, which can give rise to biased estimators.

The second is considered to be a more efficient extension of the first. It is assumed that individual effects are not reflected in the explanatory variables of the model; instead, they contribute to the error term, following the expression:

3 $${Y}_{it}= \beta {X}_{it}+\left({\alpha }_{i}+{\mu }_{it}\right),$$

where ${\alpha }_{i}$ represents the individual effects, ${\mu }_{it}$ is the error of the model, *X* would represent explanatory variables, *i* = individual and *t* = time.

The third method cited above assumes that individual effects are in line with the explanatory variables, so that the individual effect is separated after error, under the following calculation:

4 $${Y}_{it}= {\alpha }_{i}+ \beta {X}_{it}+{\mu }_{it},$$

where, again, ${\alpha }_{i}$ are the individual effects, ${\mu }_{it}$ is the error of the model, *X* are explanatory variables, *i* = individual and *t* = time.

## Results

### Land Surface Temperature by Sentinel day and night versus Rural Weather Stations

Overall, the Sentinel day and night products present higher mean values than those obtained from the AEMET rural meteorological stations for the study periods in 2019 and 2020. The two temperatures are different but correlated, meaning they will serve later to validate the LST data obtained by satellite. Specifically, in the morning the highest mean LST values are obtained using the official product Sentinel day (39.46 °C), while the mean environmental temperature of the rural station was lower (35.87 °C). At night, the highest mean LST values are obtained with the official Sentinel product (24.05 °C), and the mean environmental temperature of rural stations was again lower (21.10 °C). The mean differences obtained between the LSTs with satellite images and the rural stations amounted to 3.59 °C for Sentinel Day, and 2.95 °C for Sentinel night. Findings of increased LST with Sentinel-3 images are reproduced for both inland cities and coastal cities: the former show LST differences of 3.70 °C with Sentinel day and 3.10 °C with Sentinel night, while coastal cities show LST differences of 3.48 ºC with Sentinel day and 2.84 °C with Sentinel night.

### LST Amplified Under Heat Waves

**Fig. 3:**
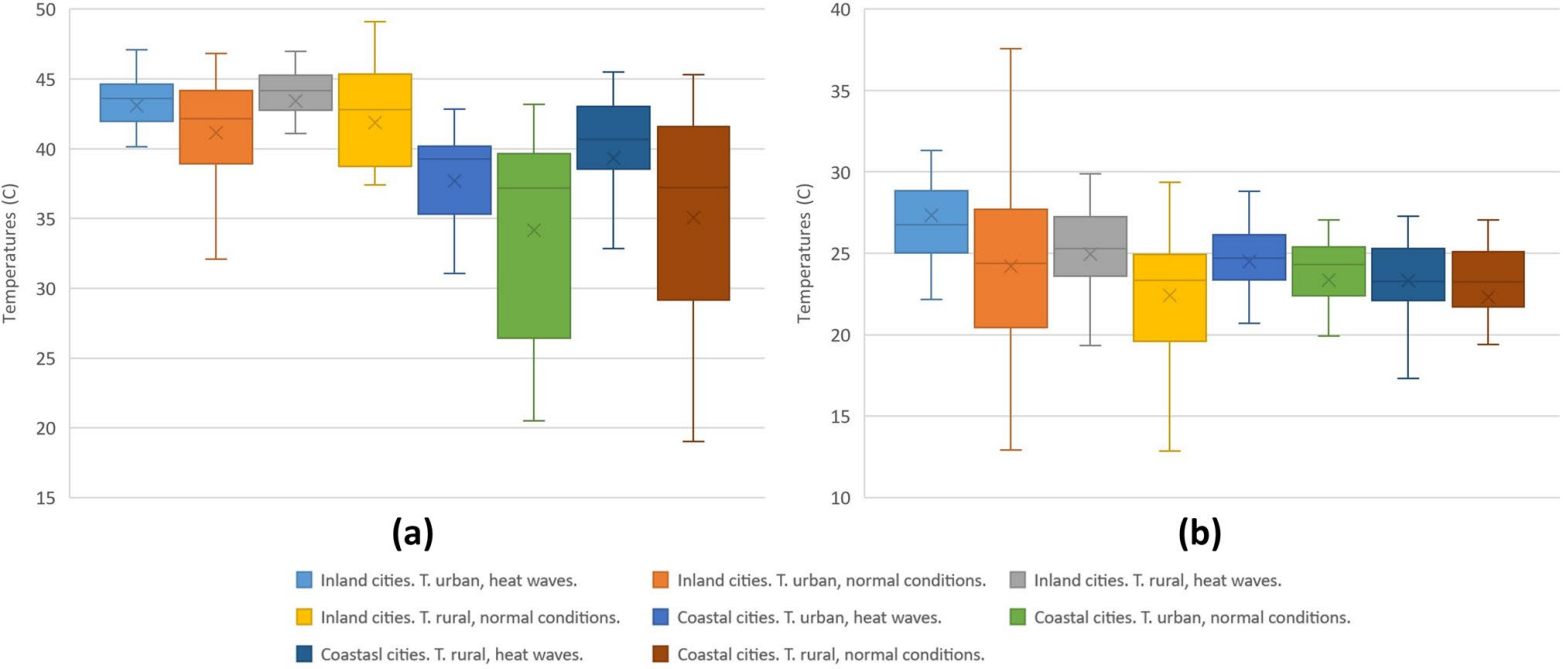
LST Sentinel Day (**a**) and night (**b**) by city type and during the period under study

The statistics of the daytime and nighttime LST obtained by means of the Sentinel day and night products for the inland and coastal Andalusian cities during the period under study are shown in Fig. 3. As can be seen, the daytime LSTs of the inland cities are higher than the LSTs of the coastal cities, whether under normal environmental conditions or in periods of heat wave. The nighttime LSTs of inland cities are seen to be lower than those of coastal cities, both under normal environmental conditions and during heat waves.

As can be seen in Table 4, during the mornings the LST values of urban areas are lower than the values of rural areas. Numerous academic studies (Saaroni et al. 2018; Wu et al. 2019; Yang et al. 2020a) indicate that the reasons for the higher LST in rural areas is motivated by the higher long wave radiation received by rural areas compared to urban ones, owing to the shade generated by buildings and trees and the cooling rates produced in urban green areas. The increases in diurnal LST in inland cities under heat wave conditions with respect to the same areas in normal conditions were 1.93 °C and 1.55 °C for urban and rural areas, respectively. In contrast, the nocturnal temperature differences respectively amounted to 3.15 °C and 2.54 °C. The diurnal LST increase in coastal cities in heat wave conditions with respect to the same areas in normal conditions was 3.53 °C for urban and 4.26 °C for rural areas. The nocturnal increases gave values of 1.13 °C and 1.02 °C, respectively.

**Table 4:** LST results with Sentinel day and Sentinel night urban and rural areas

	Daytime normal conditions		Daytime heat waves		Nighttime normal conditions		Nighttime heat waves	
Zones	Urban	Rural	Urban	Rural	Urban	Rural	Urban	Rural
Inland cities	41.13	41.87	43.06	43.42	23.37	22.31	24.19	22.40
Differences	0.74		0.36		1.06		1.79	
Coastal cities	34.15	35.05	37.68	39.31	24.50	23.33	27.34	24.94
Differences	0.90		1.63		1.17		2.40	

Temperatures: ºC

In view of the above results, it can be said that periods of heat wave entail increases in the day and night LSTs for both urban and rural areas, in the coastal as well as the inland cities of Andalusia. Still, the increase is greater during the morning in the coastal cities, and in the afternoon in the inland cities (Table 4). During the morning, the coastal cities present average values that are 3.90 °C higher when compared to the periods of normal environmental conditions; the increase in LST produced in the inland cities is, in contrast, only 1.74 °C. Contrariwise, at night, the coastal cities present mean values 1.08 °C higher than the values for periods of normal environmental conditions, as opposed to the increase in LST produced in the inland cities of 2.85 °C.

### SUHI Amplified Under Heat Waves

**Fig. 4:**
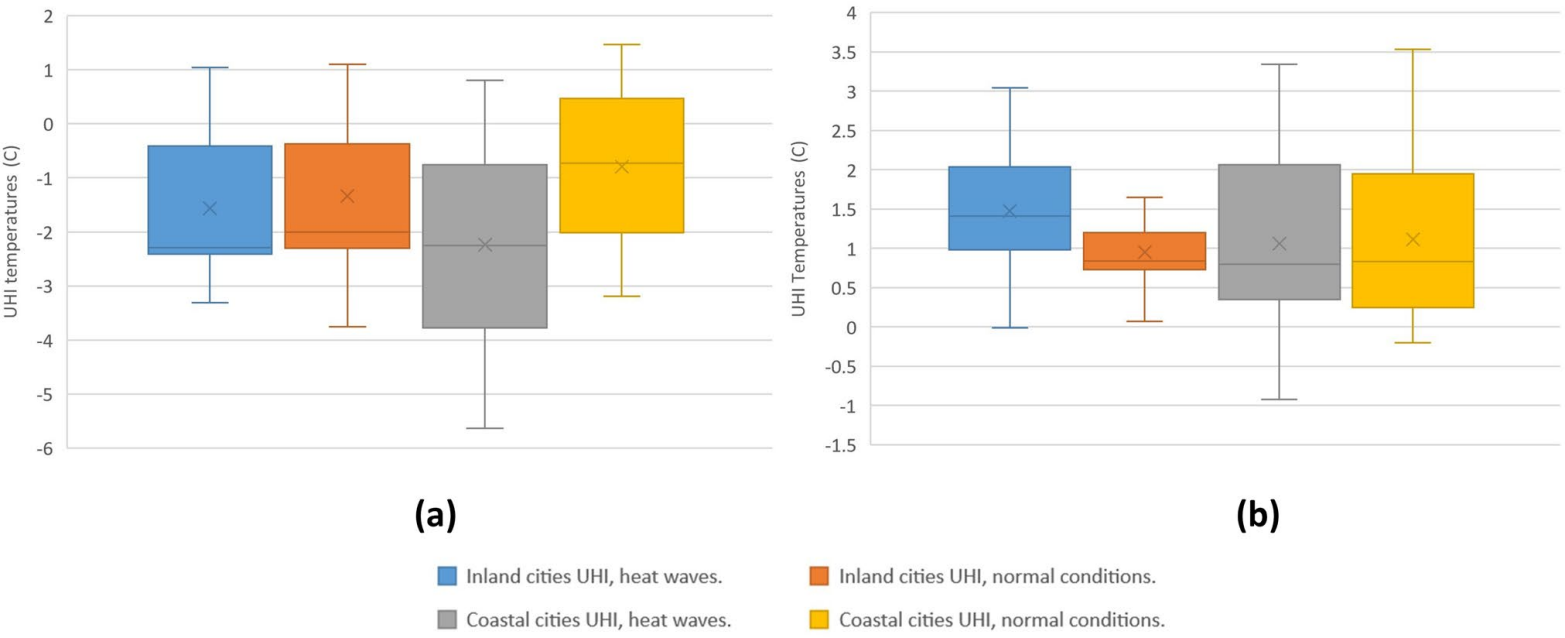
SUHI Sentinel Day (**a**) and night (**b**) by city type during the period under study

The statistics of the diurnal SUHI obtained with day and night Sentinel products for the inland and coastal cities during the study period are shown in Fig. 4.

As Table 5 shows, the cities of Andalusia present negative mean values for the diurnal SUHI that intensify in heat wave conditions, most notably in coastal cities. Similarly, the night SUHIs present positive mean values, intensified under heat wave conditions. However, their intensification is greater in inland cities than in coastal cities. The negative values indicate that during the morning, temperatures in rural areas are higher than temperatures in urban areas, producing the phenomenon known as urban cooling island (Saaroni et al. 2018; Wu et al. 2019; Yang et al. 2019). In the early morning hours, solar radiation is greater in rural areas because in the city, shade is generated by buildings, trees, and the heterogeneous system of impermeable walls with great thermal absorption and heat capacity. The sources of shade in the city prevent long wave solar radiation from heating the waterproof walls of urban areas and giving off high doses of heat, altering the LST of the area (Li and Meng 2018; Lemus et al. 2020; Logan et al. 2020; Yang et al. 2020b). The areas where the rural stations are located are farmlands with less than 10% of impervious surfaces and a mean NDVI that ranges between 0.2 and 0.5.

**Table 5:** SUHI results with Sentinel day and night, urban and rural areas

Cities	Daytime normal conditions	Daytime heat waves	Nighttime normal conditions	Nighttime heat waves
Inland cities	− 1.33	− 1.56	0.95	1.47
Differences	0.23		0.52	
Coastal cities	− 0.80	− 2.24	1.06	1.11
Differences	1.44		0.05	

Temperatures: °C

**Fig. 5:**
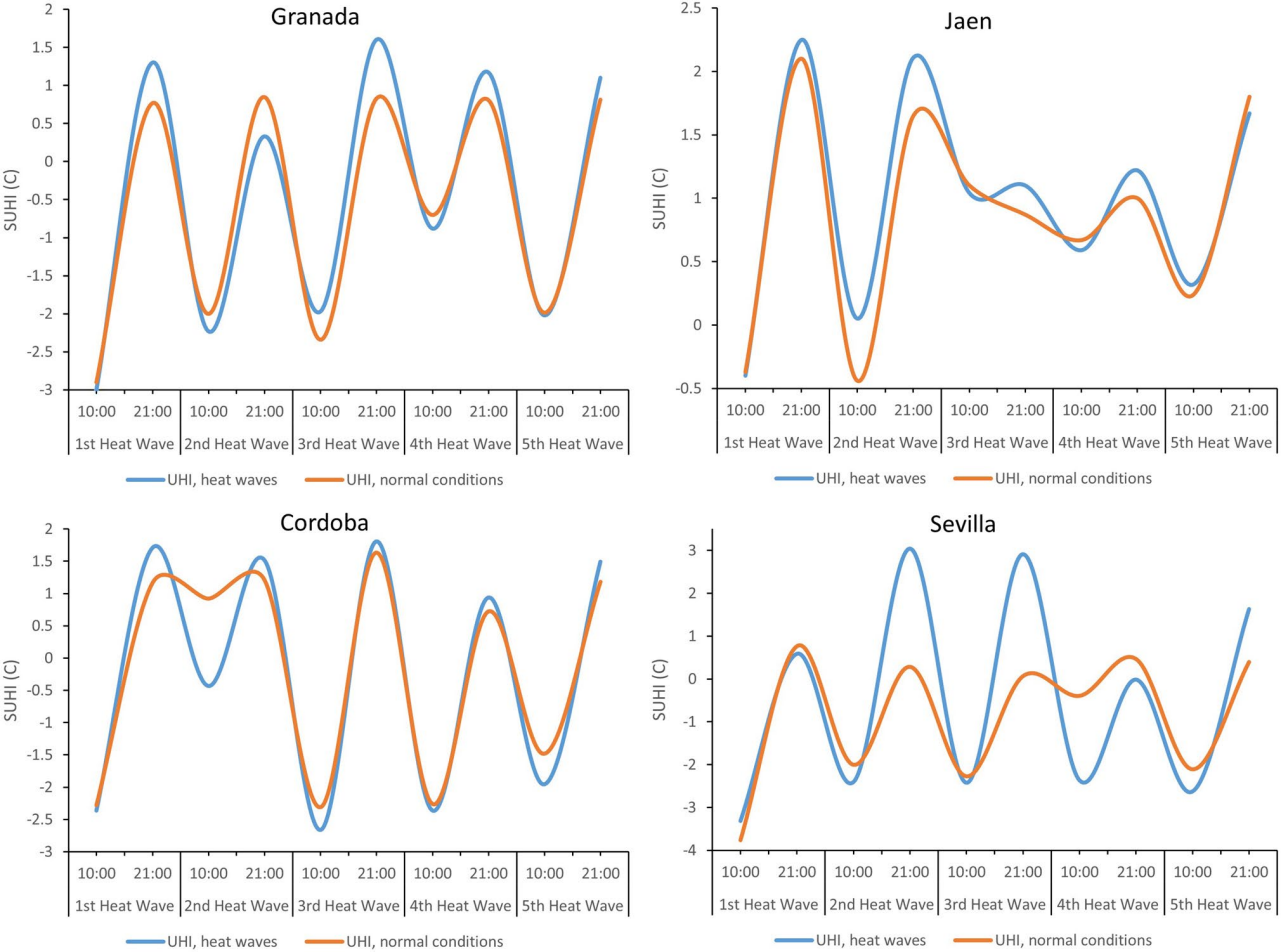
Average SUHI in inland cities under normal conditions and under heat wave, according to Sentinel day and night

**Fig. 6:**
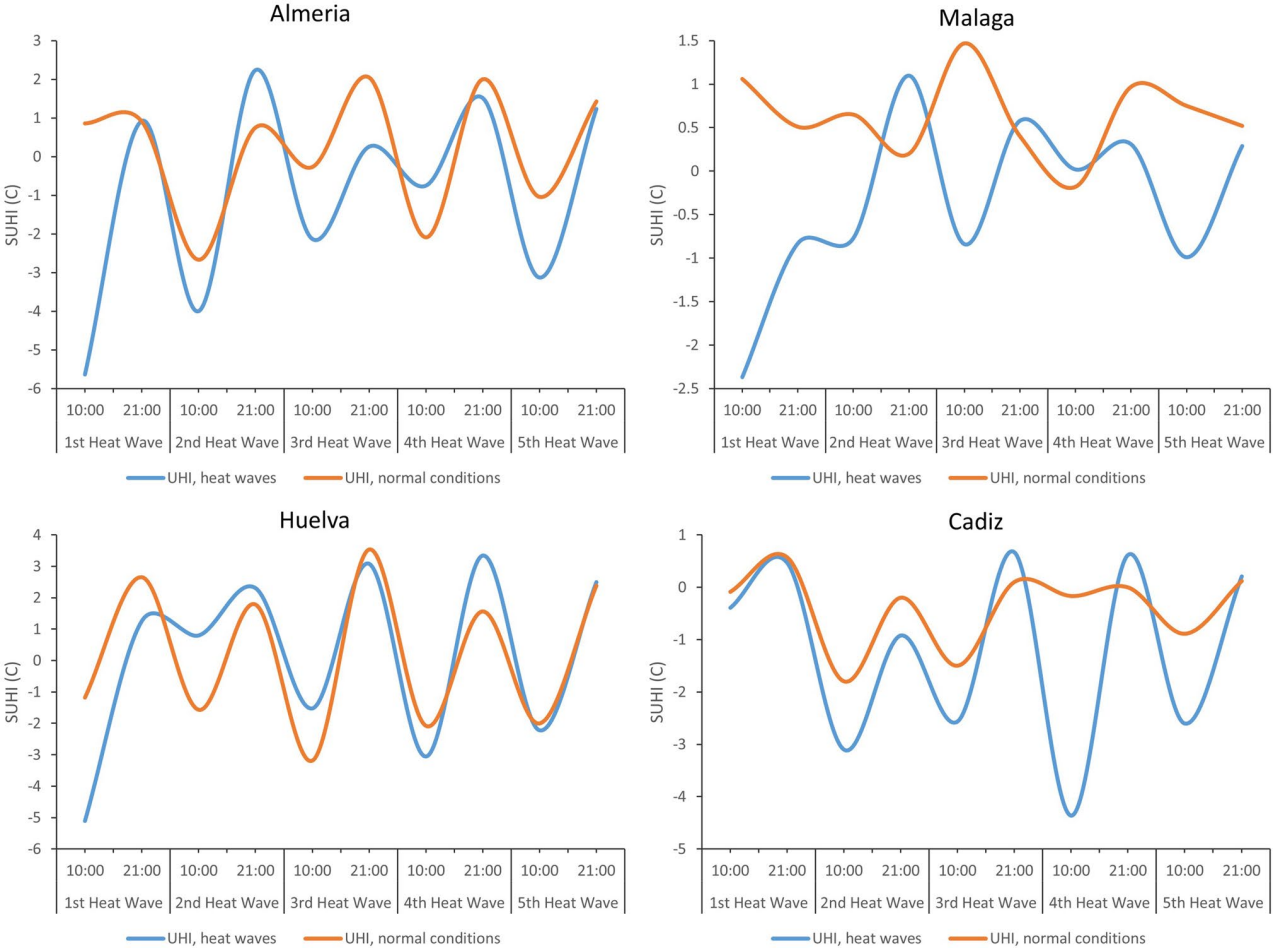
Average SUHI in coastal cities under normal conditions and under heat wave, according to Sentinel day and night

Figures 5 and 6 illustrate the diurnal and nocturnal SUHI values under heat wave conditions (blue line) and under normal environmental conditions (brown line). In general, the blue lines are found above the brown lines, indicating that the temperatures in heat wave periods are higher than the values under normal conditions. These increases occur in both inland and coastal cities. Yet the increases are greater during the day in coastal cities, and at night in inland cities.

**Fig. 7:**
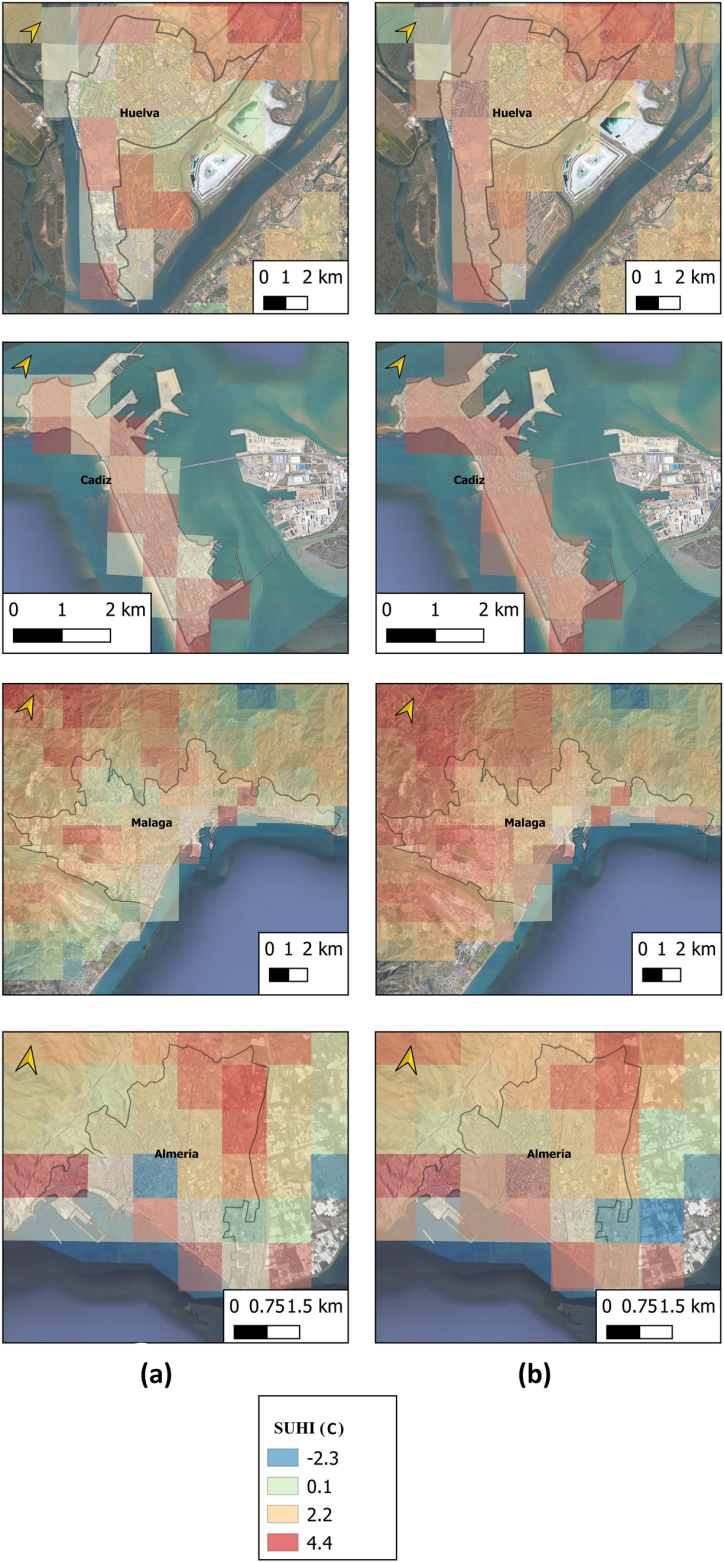
Mean SUHI during period under study for coastal cities in **a** normal environmental conditions, and **b** heat wave conditions

Figure 7 shows the mean SUHI during the period under study obtained with Sentinel day for coastal cities under normal environmental conditions and under heat wave conditions. The intensity and extension of the SUHI are seen to be greater in the images obtained during the heat wave.

**Fig. 8:**
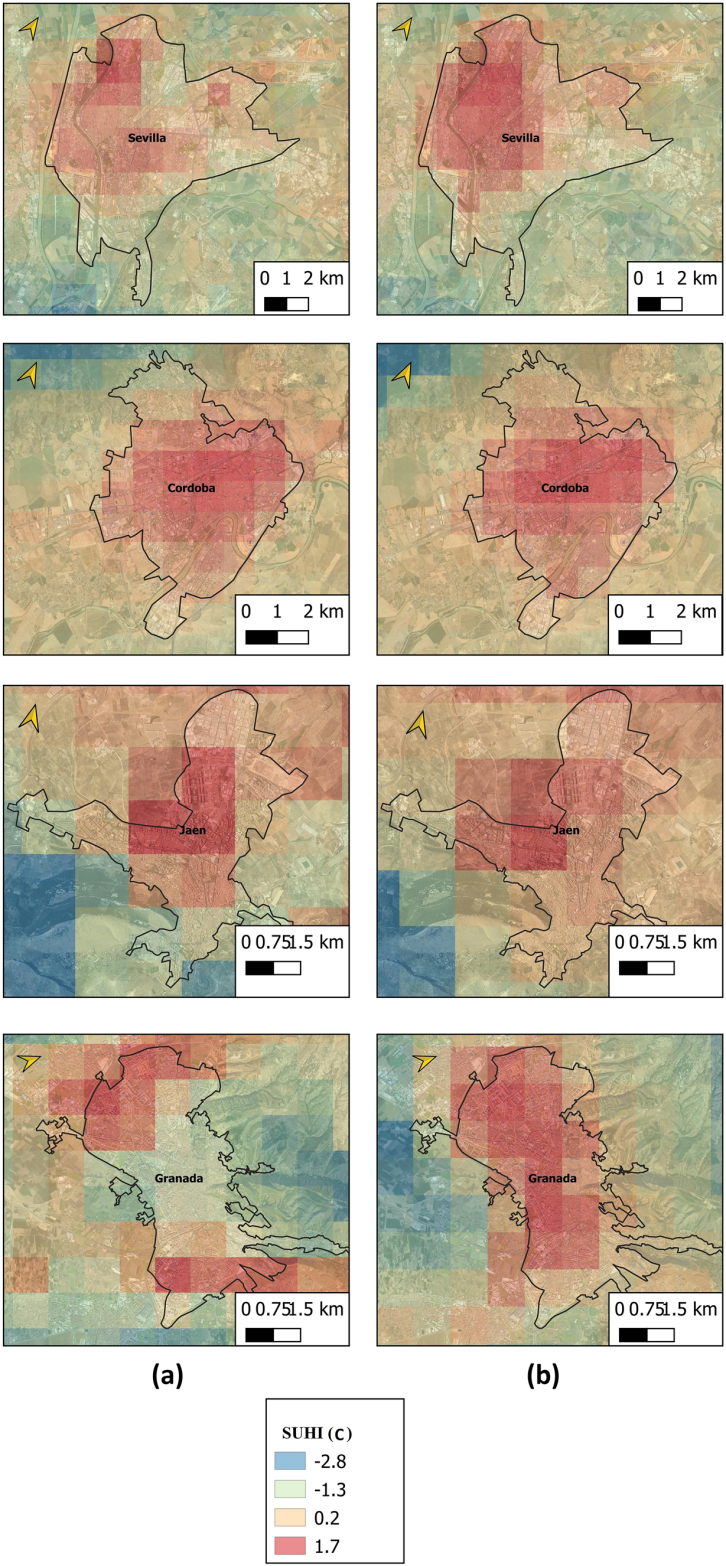
Mean SUHI during period under study for inland cities in **a** normal environmental conditions and **b** heat wave conditions

Figure 8 shows the mean SUHI during the period under study obtained with Sentinel night for inland cities under both normal environmental conditions and heat wave conditions. Both the intensity and the extension of the SUHI are greater in the images obtained during the heat wave.

### Statistical Analysis

#### Satellite Temperature Validation

To validate the satellite data obtained, it is important to obtain the linear adjustment coefficients *R*^2^, correlation coefficient (CC), standard deviation (SD), the mean bias error (MBE) and the root mean square error (RMSE), each indicated in Table 6. The results of *R*^2^ are considered adequate since they present values above 0.94. This circumstance indicates good concordance between the values analyzed, being above 94% and considered statistically significant.

**Table 6:** Data panel results for Sentinel: relationships between SUHI and LST

Satellite	CC	*R*^2^	SD	MBE	RMSE
Sentinel day	0.9970	0.95	8.57	− 4.10	2.92
Sentinel night	0.9902	0.97	6.36	− 3.85	3.18

*CC* correlation coefficient, *R*: linear adjustment, *SD* standard deviation, *MBE* mean bias error, *RMSE* root mean square error

Because these values denote a good agreement between the environmental temperature values and the LST obtained from the satellite, they lend validity to the results obtained.

Next, the statistical analysis of the variables that could influence the SUHI obtained with Sentinel day and night was carried out using the Data Panel method. The variables were: solar radiation, wind speed, and wind direction. It was first necessary to determine whether calculation should be carried out using fixed or random effects. The Hausman test was implemented to this end, its results marking the need to use the panel of robust random effects for the data obtained in the first and in the second phase of analysis. To develop the Data Panel, the Generalized Least Method (GLM) was used, with Eq. 3.

#### Interaction between SUHI and LST

The results of the statistical analysis of the LST and SUHI data obtained by Sentinel day and night in the study periods are given in Table 7.

**Table 7:** Data panel results for Sentinel: relationships between SUHI and LST

Satellite	Sentinel day			Sentinel night		
Variables	β	ρ	SD	β	ρ	SD
LST	− 0.0904	0.031	0.0419	− 0.0954	0.035	0.4538

*β* Constant, *SD* standard deviation, *ρ* *P* value

The results of the statistical analysis of the SUHI data obtained through Sentinel day and night images point to a statistically significant relationship of 95% with the independent variable LST. The values obtained for *R*^2^ and the *F* statistic of the SUHI data are shown in Table 8. The data show good agreement between the dependent and independent variables according to the method used, with a level of adjustment lower than 90% significance, as Prob > chi^2^ > 0.000.

**Table 8:** *R*^2^ and F SUHI statistical analysis for Sentinel: relationships with LST

Satellite	Sentinel day			Sentinel night		
Variables	*R*^2^	*F*	Prob > chi^2^	*R*^2^	*F*	Prob > chi^2^
LST	0.55	4.66	0.0031	0.69	4.42	0.0035

*R2* linear adjustment, *F* F statistic

### Solar Radiation Contributions to the SUHI

The AEMET has certified points for the measurement of direct and diffuse solar radiation at rural meteorological stations. Direct solar radiation is obtained by means of a Kipp-Zonen Pyrheliometer, while for diffuse solar radiation a Kipp-Zonen Pyranometer is used, periodically calibrated against international standards. The solar radiation of the rural stations of the AEMET were analyzed for the purposes of our study to grasp its influence on the variability of SUHI intensity in the cities of Andalusia. The data obtained reflect that total daily radiation is some 1.2 times higher in heat wave periods than under normal conditions. This ratio is reduced to 1.05 times in the LST and SUHI data collection chart based on Sentinel day, and up to 1.08 times higher in charts corresponding to Sentinel night. The atmospheric pressure during the periods of heat wave was 1.3 times higher than under normal conditions. These results suggest that Andalusia tends to have higher atmospheric pressures associated with less cloud cover during heat wave days, which allows more solar radiation to reach the earth's surface, as brought out in other studies (De Boeck et al. 2010; Li et al. 2015; Jiang et al. 2019).

The results of the statistical analysis of the SUHI data obtained by Sentinel day and night in periods of normal conditions and in heat waves with regard to solar radiation are indicated in Table 9.

**Table 9:** Data panel results for Sentinel: relationships with solar radiation

Satellite	Normal conditions			Heat waves		
	β	ρ	SD	β	ρ	SD
Sentinel day	0.0739	< 0.001	0.01844	0.0834	< 0.001	0.00234
Sentinel night	0.0062	0.002	0.00203	0.0075	< 0.001	0.00058

*β* Constant, *SD* standard deviation, *ρ* *P* value

The results of the statistical analysis of the SUHI data obtained through Sentinel day images point to a statistically significant relationship above 99% with the independent variable solar radiation, both under normal atmospheric conditions and in heat wave periods. The results of the statistical analysis of the SUHI data obtained through Sentinel night images indicate a statistically significant relationship of 99% with the independent variable solar radiation during periods of normal atmospheric conditions, and above 99% during heat waves. The cities are located in latitudes where the sunset during the summer period is after the time of the Sentinel night.

The values obtained for *R*^2^ and the *F* statistic of the SUHI data for Sentinel day and night are shown in Table 10. The data are seen to show good agreement between the dependent and independent variables according to the method used, with an adjustment level of 99% significance, since Prob > chi^2^ < 0.000. The *R*^2^ and *F* values are slightly higher for heat wave conditions than for normal environmental conditions, which denotes that the relationship between SUHI and solar radiation is stronger during heat waves.

**Table 10:** *R*^2^ and F SUHI statistical analysis for Sentinel: relationships with solar radiation

Satellite	Sentinel day			Sentinel night		
	*R*^2^	*F*	Prob > chi^2^	*R*^2^	*F*	Prob > chi^2^
Normal conditions	0.71	16.06	0.0004	0.72	9.42	0.0009
Heat waves	0.72	18.50	0.0001	0.75	166.39	0.0000

*R2* Linear adjustment, *F* F statistic

#### Wind Speed and Direction Contributions to the SUHI

The wind speed and wind direction data from the rural stations of the AEMET were analyzed to discern their influence on the variable intensity of the SUHI in the cities of Andalusia. The direction under normal conditions is from sea to inland (northward), while during heat wave environmental conditions, the predominant wind direction is from land to sea (southward). This means that during the day, in heat wave conditions, the cool breeze from the sea—which is usual in normal environmental conditions—does not reach cities, so that the LST and SUHI increase. The change in wind direction therefore has a strong impact on the ambient temperature.

Our data reflect a mean daytime wind speed during data collection using Sentinel day was approximately 1.16 times higher during periods of normal conditions than during heat waves. This proportion increases slightly, to 1.18 times, using Sentinel night.

Tables 11 and 12 offer the results of statistical analysis of the SUHI data obtained with Sentinel day and night in normal conditions versus periods of heat wave in terms of wind speed and direction. The analysis of SUHI data obtained with Sentinel day and night images gives no statistically significant relationship among the variables analyzed for periods of normal conditions. In contrast, with Sentinel day and under heat wave conditions there is a statistically significant relationship above 99% with the variable wind direction, slightly reduced to just 99% by Sentinel night.

**Table 11:** Data panel results Sentinel day: relationship with wind speed and direction

Satellite	Sentinel day, normal conditions			Sentinel day, heat waves		
	β	p	SD	β	p	SD
Wind speed	0.0381	0.636	0.08068	− 0.0562	0.316	0.05614
Wind direction	− 0.0015	0.497	0.00231	− 0.0053	< *0.001*	0.00146

*β* Constant, *SD* Standard deviation,* p* *P* value

*p* value is indicated in italics

**Table 12:** Data panel results Sentinel night: relationship with wind speed and direction

Satellite	Sentinel night, normal conditions			Sentinel night, heat waves		
	β	p	SD	β	p	SD
Wind speed	− 0.0651	*0.049*	0.03315	− 0.0234	0.936	0.2913
Wind direction	0.0028	0.244	0.00246	− 0.0057	*0.005*	0.0021

*β* Constant, *SD* standard deviation, *p* *P* value

*p* value is indicated in italics

The values obtained for *R*^2^ and the *F* statistic of the SUHI data from Sentinel 3A and 3B are shown in Table 13.

**Table 13:** R^2^ and F SUHI for Sentinel: relationship with wind speed and direction

Satellite	Sentinel day			Sentinel night		
	*R*^2^	*F*	Prob > chi^2^	*R*^2^	F	Prob > chi^2^
Normal conditions	0.65	3.72	0.1555	0.71	4.30	0.1167
Heat waves	0.69	13.44	0.0009	0.75	8.90	0.0007

*R*^*2*^ Linear fitting coefficient, *F* F statistic

The data gathered in periods of normal conditions do not show good agreement between the dependent variable and the independent variables according to the method used, with a level of adjustment lower than 90% significance, as Prob > chi^2^ > 0.000. On the contrary, for periods of heat wave, method used indicates good agreement between the dependent variable and the independent variables, with an adjustment level greater than 99% significance, Prob > chi^2^ < 0.000. The *R*^2^ and *F* values are slightly higher in heat wave conditions than in normal environmental conditions, which denotes that the relationship between SUHI and wind speed and direction is stronger during heat wave periods.

#### Increase in the Surface Affected by SUHI During Heat Wave Period

During heat waves, there is not only an increase in the LST and an intensification of the SUHI of the analyzed cities, but larger urban areas are reportedly affected by the SUHI as well. The average increase in these terms during the heat wave periods studied was 15.66% for the urban areas of the cities of Andalusia.

**Fig. 9:**
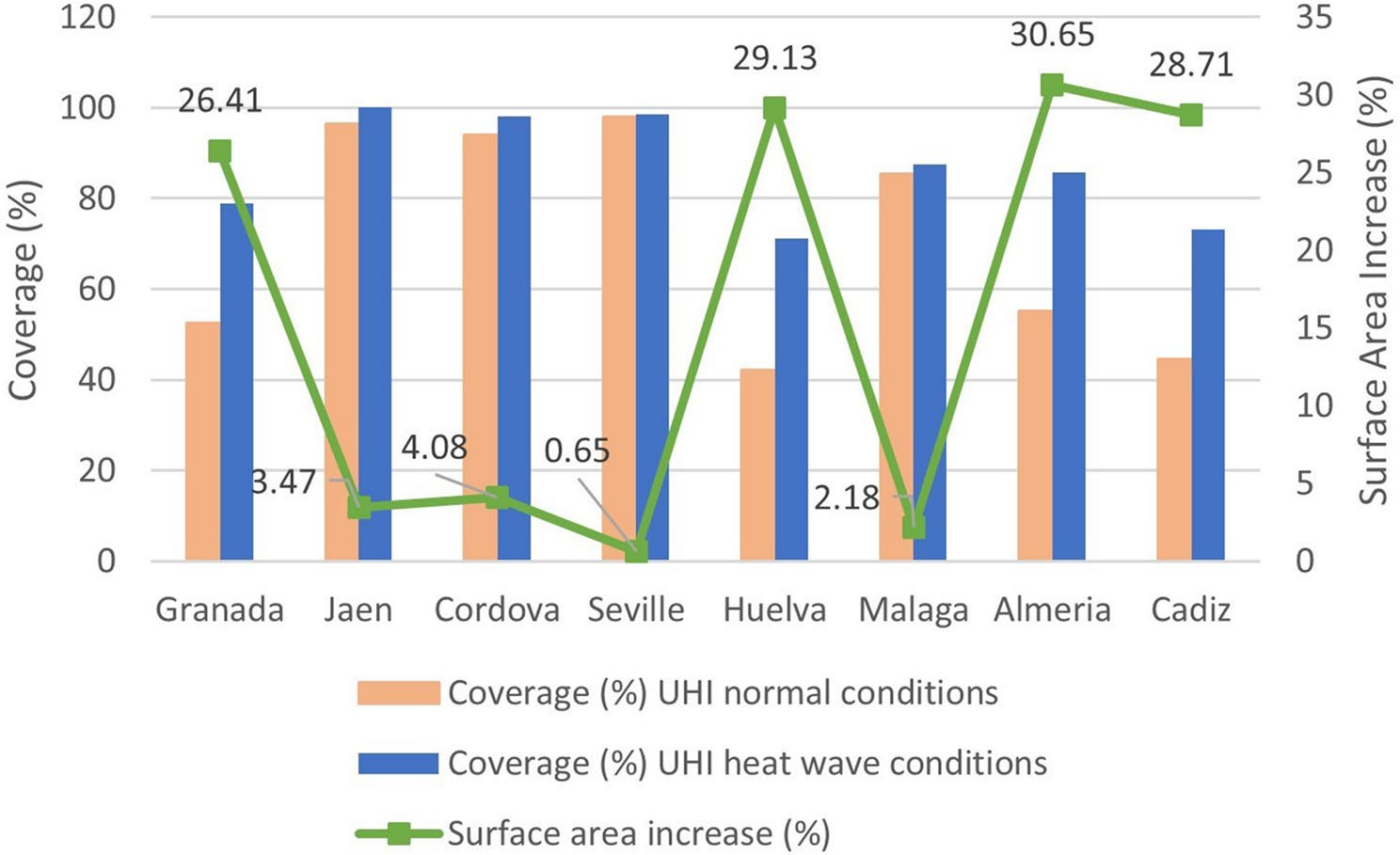
Surface area increases under normal conditions and under heat waves, and increase in urban area by cities

A substantially greater increase is observed in coastal cities (22.67%) than in inland cities (8.65%). Figure 9 shows the affected urban area under normal environmental conditions, under heat wave conditions, and the increase in the urban area of each city. It should be noted that, in general, inland cities have larger urban areas affected by SUHI under normal conditions (85.23%) than coastal cities (56.76%). This circumstance is possibly motivated by the direction of the wind, from the sea and towards the land (northward), which minimizes the LST in the latter cities. Under heat wave conditions, inland cities also present greater total urban areas affected (93.88%) than coastal cities (79.43%), although the highest growth of SUHI occurs in coastal cities. The change in wind direction (southward) can be considered the reason for this finding.

## Discussion

The results presented here, for Sentinel day and night products used to obtain the LST and the SUHI in the cities of Andalusia, present adequate yields that are consistent with each other and similar to those provided by similar investigations (Li et al. 2011; Tan and Li 2015; Sobrino et al. 2016; Prikaziuk and van der Tol 2019; Yang et al. 2019, 2020b; Chiang and Ivan 2020; Hu et al. 2020; Venter et al. 2020).

The data obtained with Sentinel day, both for inland cities and coastal cities, give mean LSTs in rural areas that are higher than the mean LSTs in urban areas, both in periods of normal environmental conditions and in periods of heat wave. Unlike Sentinel day, Sentinel night data report that both inland cities and coastal cities have mean LSTs in rural areas that are lower than the mean LSTs of urban areas, whether under normal conditions or in heat waves. There are numerous academic studies that corroborate this situation between urban and rural temperatures in the early hours of the morning and at night, motivated by the solar radiation received (Zakšek et al. 2005; Keramitsoglou et al. 2011; Li et al. 2011; Feizizadeh and Blaschke 2013; Li and Bou-Zeid 2013; Mallick et al. 2013; Founda and Santamouris 2017; Tsou et al. 2017; Barbieri et al. 2018; Li and Meng 2018; Saaroni et al. 2018; Karakuş, 2019; Wu et al. 2019; Yang et al. 2019, 2020a, b; Lemus et al. 2020).

The mean values of SUHI obtained through Seninel day images for inland and coastal cities were negative. This finding, likewise evoked by other authors, would be determinant of an urban cooling island (Saaroni et al. 2018; Wu et al. 2019; Yang et al. 2020a). In turn, the mean SUHI data obtained by Sentinel night for inland and coastal cities were positive—indicative of an urban heat island, a phenomenon previously studied (Li et al. 2011; Shwarz et al. 2011; Lai et al. 2018; Luo and Lau 2018; Zhao et al. 2018; Tewari et al. 2019; Anjos et al. 2020; Huang et al. 2020; Santamouris, 2020). In light of our data, it can be said that during periods of heat waves there is an intensification of the SUHI obtained by Sentinel day and night, both in inland cities and in coastal cities. However, this intensification is greater with Sentinel day in coastal cities, and with Sentinel night in inland cities. Numerous academic studies corroborate the intensification of the SUHI at night (Gregor et al. 2007; Basara et al. 2010; House and Santamouris 2011; Founda et al. 2015; Jiang et al. 2019;) and during the day (House and Santamouris 2011; Founda and Santamouris 2017; Ao et al. 2019; Jiang et al. 2019; Qiu et al. 2020; Santamouris 2020) in periods of heat wave.

The data on total daily solar radiation obtained attest to a 1.2-times increase in periods of heat wave with respect to normal conditions, corroborated by statistical analysis. A number of academic studies confirm this association between solar radiation and SUHI (De Boeck et al. 2010; Li et al. 2015; Li and Bou-Zeid 2013; Jiang et al. 2019), serving to validate the data obtained in our investigation.

The wind speed and direction data gathered in our study denote important changes in the cities of Andalusia between periods of normal environmental conditions and periods of heat wave, corroborated by statistical analysis. The relationship between SUHI and wind speed and direction are stronger during heat wave periods. Numerous studies describe such an intensification of the SUHI in the early hours of the morning and at night (Ackerman and Knox 2012; Li and Bou-Zeid 2013; Li et al. 2015; Ramamurthy and Bou-Zeid 2017; Jiang et al. 2019; An et al. 2020), thus validating the data obtained in this investigation.

According to our data, an average urban area in southern Spain would be affected by the SUHI phenomenon under normal environmental conditions to the extent of 85.23% in inland cities and 56.76% in coastal cities. The average urban surface affected by the SUHI phenomenon under heat wave conditions would be 15.66% greater, when compared to periods of normal environmental conditions. Still, this increase is uneven: 22.67% for coastal cities and 8.65% for inland cities. Research by other authors (Lemonsu et al. 2015; Ward et al. 2016; Carvalho et al. 2017; Jiang et al. 2019) presenting similar values comes to support the results obtained here.

## Conclusions

In this work, the LST and SUHI were studied by analyzing Sentinel day and night images of the eight capitals of Andalusia (southern Spain) both in periods of normal environmental conditions and in periods of heat wave, during the years 2019 and 2020. A statistically significant relationship between the two variables is evidenced.

Our results detect mean LSTs based on Sentinel day and night in inland cities—both under normal environmental conditions and in periods of heat wave—that are higher than the mean LSTs of coastal cities. In turn, the average LSTs obtained with Sentinel day and night products for both urban and rural areas are intensified under heat wave environmental conditions, the increase being greater with Sentinel day in coastal cities, and with Sentinel night in inland cities.

The mean SUHI obtained with Sentinel day during the entire study period for the capitals of the Andalusian provinces showed negative values, whereas the mean SUHI obtained with Sentinel night showed positive values. This suggests that urban areas are at lower temperatures in the morning than neighboring rural areas, a phenomenon known as urban cooling island. Then, during the evening, the urban areas are at higher temperatures than the adjacent rural areas, producing an urban heat island. During heat wave periods, an intensification of the SUHI obtained with Sentinel day and night is detected for both inland cities and coastal cities, but it is greater for coastal cities with Sentinel day, and for inland cities with Sentinel night.

Within the scope of the environmental factors studied, our results attest to a positive and statistically significant relationship between SUHI and solar radiation, and between SUHI and the direction of the wind, intensified in periods of heat wave as compared to periods of normal environmental conditions. Wind speed turns out to be a positive and statistically significant variable, but only in periods of normal conditions and according to the data from Sentinel night images.

Our results detect that the surface of the urban area affected by the SUHI phenomenon under normal environmental conditions is greater for inland cities than for coastal cities. Notwithstanding, under heat wave conditions, the intensified SUHI entails a larger surface area, this phenomenon being greater for coastal cities than for inland cities.

## Supplementary Information

Below is the link to the electronic supplementary material.

Supplementary file1 (PDF 391 kb)

## Data Availability

Not applicable.
