# Supplementary material for: Analysis of Urban Heat Island and Heat Waves Using Sentinel-3 Images: a Study of Andalusian Cities in Spain
Source: Earth Syst Environ. 2021 Nov 2;6(1):199–219. doi: 10.1007/s41748-021-00268-9 (PMC8562036; doi:10.1007/s41748-021-00268-9)
Supplement: Supplementary file 1 — Supplementary file1 (PDF 391 kb) [file 41748_2021_268_MOESM1_ESM.pdf]

Highlights:

1º) An intensification of LST and UHI obtained with Sentinel under heat waves is reported.

2º) An intensification of synergies between UHI and solar radiation is reported.

3º) An intensification of the synergies between UHI and the wind direction is reported.

4º) The intensification of Sentinel 3A in coastal cities is: LST = 3.90 K and UHI = 1.44 K.

5º) The intensification of Sentinel 3B in inland cities is: LST = 2.85 K and UHI = 0.52 K.
